# Bioethical implications of cosmetic psychiatry: Distributive justice versus utilitarianism

**DOI:** 10.1177/10398562241264422

**Published:** 2024-06-21

**Authors:** Michael James Weightman, Andrew James Amos

**Affiliations:** Adelaide, SA; Townsville, QLD

Dear Editor,

The concept of cosmetic psychiatry described by Lugg^1^ radically departs from traditional practice. It broadens psychiatric scope from treatment of mental illness to enhancement of wellbeing. We agree that psychiatry should lead this discussion to ensure patient and community interests are maintained, but also wish to raise some important ethical considerations.

A key bioethical principle is distributive justice.^2^ Access to care is arguably the most significant challenge facing psychiatry; Australia has critical shortages of psychiatrists, particularly in rural/remote and lower socioeconomic areas.^3^ It would be ethically dubious to redirect limited psychiatrist time towards improving the performance of already advantaged people at the expense of those seeking care for mental illness. Rather, a socially just allocation of limited resources would ensure these are deployed to those in greatest need. This is especially true for publicly funded psychiatric care.

The public health imperative of ensuring scarce resources are used wisely is challenged by market forces. The wellness industry is enormously lucrative^4^; it is likely to become more financially rewarding for a psychiatrist to focus on increasing happiness in relatively happy people rather than reducing despair or dysfunction in the mentally unwell. Such a shift from treating “patients” to “consumers” would alter the current pastoral clinical role into a more commercial relationship, fundamentally changing the practice of medicine. The moral calculus here might change if the commercial energy generated by cosmetic psychiatry could be harnessed to improve outcomes for the mentally ill, for example, by increasing the psychiatric workforce more than required to address the cosmetic demand. This would be a complicated endeavour.

Lugg hints at a utilitarian counterargument by referencing the performance enhancement sought by entrepreneurs such as Steve Jobs and Tim Ferris.^1^ One could argue that significant benefits may be available to society collectively if cosmetic psychiatry interventions could enhance the output of gifted individuals (e.g., entrepreneurs/researchers) or key decision-makers (e.g., Members of Parliament). Investing resources in these privileged members of society may justify abandoning the most vulnerable if we can be assured their improved decisions or productivity would benefit the wider population. Such a situation may theoretically result in the greatest good to the greatest number, but leaves behind the least able in a way that is unlikely to be palatable for members of a profession built on aspirations for helping others.

The alternative is allowing cosmetic psychiatry to become the purview of non-psychiatrists. This would be harder to justify, as the training and expertise of psychiatrists in combination with the stringent regulatory frameworks of medical practice theoretically ensures greater protections to the public compared to a lay prescriber. A pertinent analogy may be drawn with cosmetic surgery, where unregulated practice has led to adverse outcomes and prompted moves to return cosmetic work to the surgical fraternity.^5^

To conclude, whilst proliferation of cosmetic psychiatry may prove inevitable, it risks exacerbating current inequities and undermining the long-established doctor–patient dynamic. Cosmetic psychiatry must be considered an experimental concept and requires much clearer clinical governance before progression into mainstream psychiatric practice.
